# Latent profile analysis of electronic health literacy and its impact on health promoting lifestyles among maintenance hemodialysis patients

**DOI:** 10.3389/fpubh.2025.1630350

**Published:** 2025-10-17

**Authors:** Lan Yang, Jinghua Yang, Hong Zhang, Ning Wu, Xuejiao Han, Na Li

**Affiliations:** ^1^Department of Nephrology, Baoding No. 1 Central Hospital, Baoding, Hebei, China; ^2^Internal Medicine-Cardiovascular Department, Dongzhimen Hospital of Beijing University of Chinese Medicine, Beijing, China

**Keywords:** e-health literacy, maintenance hemodialysis, health-promoting lifestyle, latent profile analysis, quality of life

## Abstract

**Background:**

Despite the critical role of e-Health literacy (eHL) in modern healthcare, current research predominantly concentrates on conditions such as cancer and diabetes, as well as outpatient care settings. However, there remains a significant gap in studies specifically addressing the eHL needs of patients with maintenance hemodialysis (MHD).

**Objective:**

This study aims to explore the latent categories of eHL among MHD patients and its impact on health-promoting lifestyle (HPL).

**Methods:**

A survey was conducted using a convenience sampling method involving 500 MHD patients from three tertiary hospitals in Baoding. Data were analyzed using latent profile analysis (LPA) and a mixed regression model.

**Results:**

This study showed that MHD patients could be classified into low (23.17%), middle (49.78%), and high (27.05%) eHL groups, with the three-class model showing optimal fit (AIC = 2321.213, BIC = 2271.168, entropy = 0.967). MHD Patients in the high literacy group scored significantly higher in all dimensions of e-HL and overall HPL (119.58 ± 13.86) compared to those in the low literacy group (91.82 ± 11.73) (all *p* < 0.001). Multivariate analysis identified high educational level (OR = 1.512), higher average monthly household income (OR = 1.511), longer dialysis duration (OR = 1.314), and self-management abilities (OR = 1.243) as independent predictors of eHL.

**Conclusion:**

The findings suggest a heterogeneous stratification of eHL among MHD patients, closely linked to HPL. Stratified intervention strategies should be developed for different patient groups to potentially improve their health behaviors. The study provides evidence-based support for personalized health management.

## 1 Introduction

Electronic health literacy (eHL) is an emerging concept, defined as the ability of individuals to seek, understand, and utilize basic health information and services via electronic networks to make informed decisions that foster and maintain good health (1). As society increasingly digitizes, this concept has been integrated into online health interventions across various diseases, allowing for quick access to health information via the internet (2). The feasibility of these interventions makes eHL a critical area of study, particularly as healthcare transitions further into the digital realm.

Maintenance hemodialysis (MHD) is the primary mode of renal replacement therapy for patients with end-stage renal disease (ESRD), with a patient population that continues to grow (3). Studies have demonstrated that MHD patients knowledgeable about monitoring their condition—notably through compliance with medication regimens, dietary and fluid restrictions, and vascular access care—exhibit reduced complications and hospitalization rates (4). High levels of eHL enable MHD patients to manage their condition more effectively, potentially improving their quality of life (QoL) (5).

There is a growing body of evidence from international studies suggesting that individuals with high eHL are more likely to engage in health-promoting behaviors, such as adopting nutritious diets, maintaining exercise routines, and ensuring adequate sleep (6–8). This indicates a direct correlation between eHL and lifestyle choices beneficial to health outcomes. For MHD patients, improving eHL may be crucial to enhancing their QoL and extending survival. Thus, renal healthcare providers must prioritize the eHL of MHD patients as part of comprehensive care strategies.

Despite the critical importance of eHL, existing research primarily focuses on cancer, diabetes, and outpatient settings, with limited studies dedicated to MHD patients (9, 10). Most existing studies have focused on describing overall eHL levels or examining correlations with isolated health outcomes, without delving into the heterogeneous latent structure of eHL—particularly in MHD patients, a population with complex information needs and heavy self-management burdens. Implementing latent profile analysis (LPA), a person-centered analytical approach, allows for categorizing individuals based on observable variables, facilitating the nuanced understanding of complex characteristics (11). By classifying the latent profiles of eHL among MHD patients, healthcare providers can accurately identify those with lower eHL and execute targeted interventions aimed at these specific groups.

This study employs LPA to investigate the latent profiles of eHL among MHD patients and assesses the impact of these profiles on patients’ health promoting lifestyles (HPL) using regression mixture models. However, given the established importance of eHL and the lack of research among MHD patients, this study aims to use LPA to explore the latent class structure of eHL in this population. We proposed the following hypotheses: Hypothesis 1 (H1): The eHL of MHD patients is not homogeneous and can be categorized into distinct latent classes (e.g., high, medium, and low literacy groups) using LPA. Hypothesis 2 (H2): Patients from different eHL latent classes will differ significantly in terms of demographic (e.g., education level, income) and clinical characteristics (e.g., dialysis vintage, self-management abilities). Hypothesis 3 (H3): The assigned eHL latent class will significantly predict the level of patients’ HPL, meaning that a higher eHL class will be associated with higher overall and sub-scale scores of HPL. By testing these hypotheses, this study aims to provide a precise theoretical and practical basis for stratified digital health interventions among MHD patients. The objective is to equip healthcare providers with the clinical guidance and reference necessary to develop personalized interventions for MHD patients, thereby enhancing health outcomes through tailored healthcare strategies.

## 2 Materials and methods

### 2.1 Research subjects

From June to December 2024, a convenience sampling method was employed to select MHD patients at three tertiary hospitals in Baoding City for a questionnaire survey. Inclusion criteria were as follows: (1) patients who met the diagnostic criteria for ESRD and had been on MHD for at least 3 months; (2) individuals aged 18 years or older; (3) individuals with experience in seeking health information online; (4) absence of psychiatric disorders that impair cognitive function; (5) individuals who provide informed consent and are willing to participate actively in the study. Exclusion criteria included any reasons preventing the completion of the questionnaire survey. This study was approved by the Medical Ethics Committee of Baoding No.1 Central Hospital (approval number: 2024199). All participants provided written informed consent.

### 2.2 Research methodology

**Figure 1:**
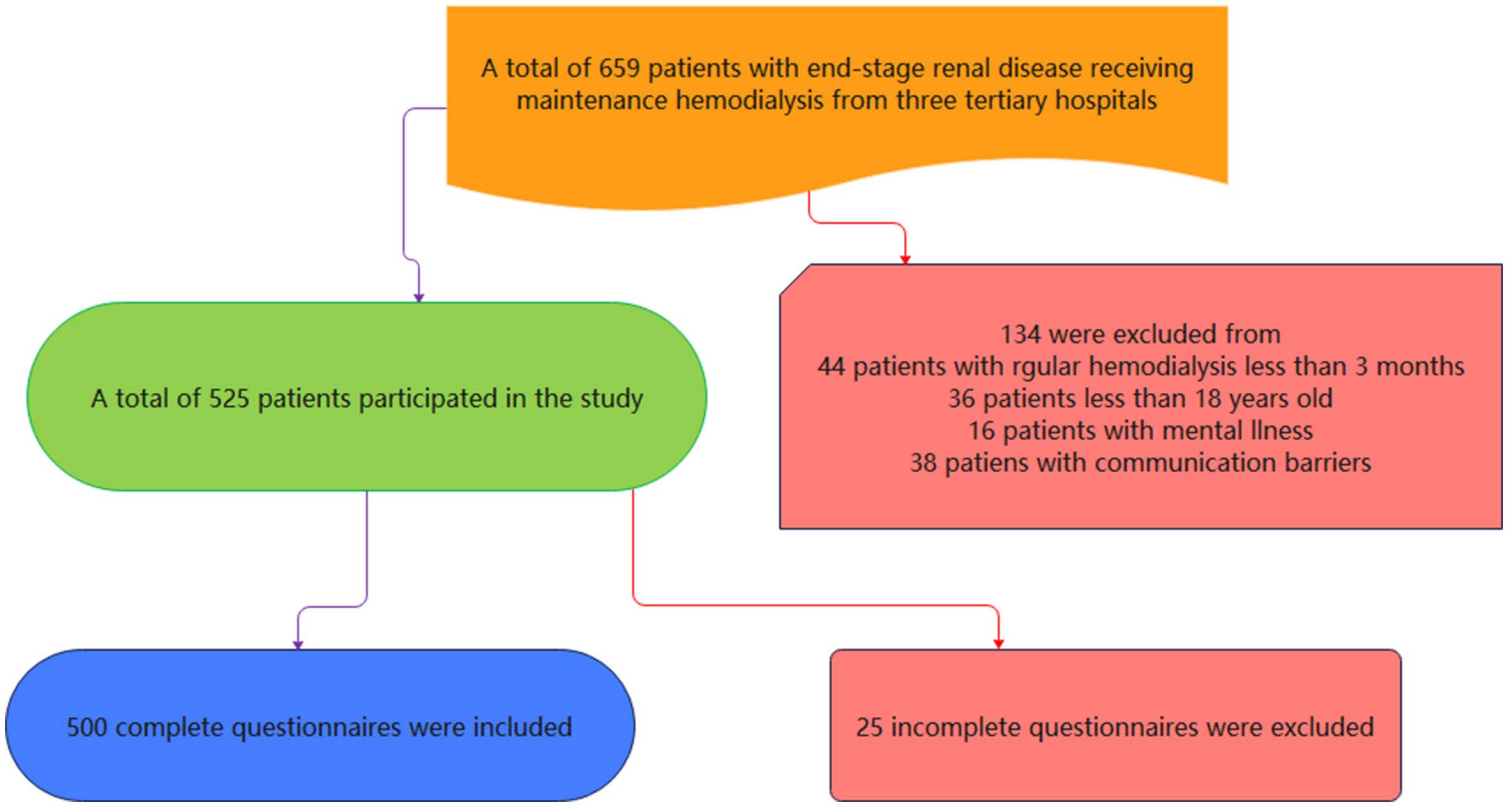
Flowchart of the study.

A single researcher was selected from each healthcare institution and was provided with standardized training on the objectives, subjects, and methods of the survey to ensure a consistent approach across all participants. Prior to the commencement of the survey, patients were informed about the purpose, procedure, and confidentiality principles of this study, and their consent was obtained before distributing the paper-based questionnaires on-site. During the completion of the questionnaire, researchers were available to promptly address any questions raised by patients. For those who found it challenging to fill out the questionnaire themselves, the researchers assisted by conducting a question-and-answer session. Once completed, the questionnaires were collected immediately and checked for completeness. Any questionnaires that were filled out in less than 5 min, responded to in a patterned way, or contained illogical answers were deemed invalid and excluded. A total of 525 questionnaires were distributed during this study, with 500 valid responses collected, resulting in an effective response rate of 95.2% (Figure 1).

### 2.3 Research instruments

The general information questionnaire was developed by the researchers to gather comprehensive demographic and personal data about the patients. This questionnaire includes details such as the patient’s sex, age, smoking and drinking habits, educational level, dialysis duration, marital status, employment status, average monthly household income, living alone, healthcare payment methods, dialysis frequency every week, complications, and self-management abilities.

The Electronic Health Literacy Scale (e-HEALS) was originally designed by Norman and Skinner and subsequently translated into Chinese by Guo Shuaijun and colleagues (12). This scale is utilized to assess the level of electronic health literacy among study participants. It is structured into three dimensions: the ability to apply online health information and services (5 items), evaluative skills (2 items), and decision-making skills (1 item), totaling 8 items. Each item is rated using a 5-point Likert scale, ranging from “strongly disagree” (1 point) to “strongly agree” (5 points). The total score spans from 8 to 40 points, with higher scores indicating a higher level of electronic health literacy. A score of 32 or above signifies adequate electronic health literacy, while a score below 32 indicates inadequate literacy. The Cronbach’s alpha coefficient for this scale is 0.967, while in the current study, it is reported as 0.782.

The Health Promoting Lifestyle Profile-II (HPLP-II) is a scale developed by Cao Wenjun and colleagues for assessing individual health-promoting behaviors (13). This instrument encompasses six dimensions: interpersonal relationships (5 items), nutrition (6 items), health responsibility (11 items), physical activity (8 items), stress management (5 items), and mental growth (5 items), totaling 40 items. Each item is scored on a 4-point Likert scale, where responses range from “never” to “always,” corresponding to scores of 1 to 4, respectively. The total score ranges from 40 to 160, with higher scores indicating a higher level of healthy behavior. In this study, the overall Cronbach’s alpha coefficient for the HPLP-II was 0.837, while the Cronbach’s alpha coefficients for the six dimensions were 0.689, 0.904, 0.827, 0.775, 0.873, and 0.829, respectively.

To ensure the scientific rigor and standardization of the investigation, the research team invited three experts, each with over 10 years of experience in the field and holding senior titles, to provide a one-week training program to three researchers. The training covered topics such as the investigation plan, procedures, guidelines and clarification for the questionnaire, and communication skills between doctors and patients. A practical assessment was conducted at the end of the training, with a score of 90 or above considered passing. Throughout the process, all three researchers were involved in the questionnaire survey and data collection; one researcher focused on administering the questionnaire, while the remaining two were responsible for organizing and verifying the data, ensuring its completeness and authenticity.

Data analysis was conducted using SPSS version 26.0. Descriptive statistics were used for categorical data, expressed as frequencies and percentages. For ungrouped categorical variables, a chi-square test was applied using contingency tables. The Kruskal-Wallis H test was utilized for analyzing ordered categorical data. Continuous data fitting a normal distribution were represented as mean ± standard deviation. Multivariate analysis was performed through multinomial logistic regression. The software Mplus version 8.3 was employed to classify the eHL of MHD patients into LPA. LPA is model-based and objectively determines the optimal number of classes using fit indices (AIC, BIC, aBIC). The entropy value assesses classification accuracy. The significance of the LMRT and BLRT tests is used to compare whether a k-class model fits significantly better than a k-1 class model. We systematically fitted models from 1 to 5 classes and determined the 3-class model as optimal because it had the lowest AIC/BIC values, the highest entropy value (0.967, close to 1, indicating excellent classification accuracy), and significant LMRT and BLRT values (*p* < 0.05). This process ensures that patient classification is not subjective but is driven by the data and determined by the optimal statistical model. Regression Mixture Model: After identifying the latent classes, we used a regression mixture model (implemented in Mplus) to examine the impact of different eHL classes on the HPL. This method is more powerful and appropriate than traditional ANOVA because it: Directly incorporates the LPA results: It uses the “posterior probabilities” of each patient’s class membership, accounting for classification uncertainty and yielding more robust results. Allows for covariate adjustment: Although this analysis primarily focused on the main effect of the class, the framework allows for the inclusion of covariates (e.g., age, gender) for adjustment in future analyses. Effectively handles the dependent variable: It models the continuous HPL scores directly, fully preserving the information in the data. Other Statistical Methods: For the univariate analysis of baseline data, we strictly selected methods based on data types: one-way Analysis of Variance (ANOVA) for normally distributed continuous variables; the Kruskal-Wallis H test for non-normally distributed continuous variables; and the chi-square test for categorical variables. Multivariate analysis was performed using multinomial logistic regression to explore independent factors influencing eHL classification, with results presented as odds ratios (OR) and 95% confidence intervals (CI). Once the optimal latent profile was determined, a regression mixture model was built using Mplus 8.3 to evaluate the impact of different eHL categories on HPL among MHD patients. A *p*-value of less than 0.05 was considered statistically significant.

## 3 Results

**Figure 2:**
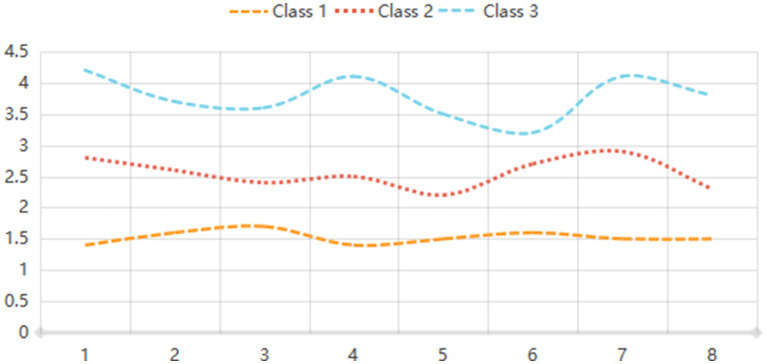
Map of e-health literacy latent profile of the participants.

LPA was conducted to identify distinct classes of eHL based on model fit indices. The analysis revealed that the three-class model demonstrated the best fit, with the lowest AIC (2321.213) and BIC (2271.168) values, along with a high entropy value of 0.967, indicating clear classification accuracy. The likelihood ratio test (LRT) and bootstrap likelihood ratio test (BLRT) further supported the three-class model (*p* < 0.01 and *p* = 0.013, respectively). The proportions of participants in the three classes were 23.17, 49.78, and 27.05%, respectively. In contrast, the two-class and four-class models showed less optimal fit, with higher AIC and BIC values and lower entropy. The five-class model was also deemed less suitable due to its higher AIC (2489.112) and BIC (2579.327) values, as well as a lower entropy value (0.922). These results suggest that eHL can be effectively categorized into three distinct profiles, providing a robust framework for further analysis (Table 1; Figure 2).

**Table 1:** Fitting results of latent profile analysis for e-health literacy.

Class	AIC	BIC	aBIC	Entropy value	*p*		Proportion (%)				
					LRT	BLRT	1	2	3	4	5
1	2437.457	2512.239	2421.276	—	—	—					
2	2342.227	2458.324	2341.173	0.912	0.143	<0.001	55.14	4.86	—	—	—
3	2321.213	2271.168	2312.214	0.967	<0.01	0.013	23.17	49.78	27.05	—	—
4	2414.113	2527.314	2436.123	0.941	0.614	0.038	21.45	35.76	21.37	21.42	—
5	2489.112	2579.327	2459.246	0.922	0.352	0.027	18.12	24.53	19.56	21.33	16.49

AIC, akaike information criterion; BIC, bayesian information criterion; aBIC, adjusted bayesian information criterion; LRT, likelihood ratio test; BLRT, bootstrap likelihood ratio test.

The comparison of eHL levels among MHD patients revealed significant differences across all dimensions and total scores (*p* < 0.001). Patients with low eHL (*n* = 116) scored the lowest in application ability (7.45 ± 1.84), evaluation ability (2.87 ± 1.26), decision-making ability (2.14 ± 0.95), and total scores (12.53 ± 4.11). In contrast, those with middle levels of eHL (*n* = 249) demonstrated higher scores in application ability (13.56 ± 4.32), evaluation ability (5.36 ± 1.64), decision-making ability (2.54 ± 1.05), and total scores (19.84 ± 4.21). Patients with high eHL (*n* = 135) achieved the highest scores in application ability (17.21 ± 3.96), evaluation ability (5.98 ± 1.52), decision-making ability (2.88 ± 1.24), and total scores (24.17 ± 3.54). These findings indicate a clear gradient in eHL levels, with significant improvements in all measured dimensions as literacy levels increase (Table 2).

**Table 2:** Comparison of total and sub-scale scores of e-health literacy among different categories of maintenance hemodialysis patients (x ± s).

Categories	*N*	Application ability	Evaluation ability	Decision-making ability	Total scores
Low levels of e-health literacy	116	7.45 ± 1.84	2.87 ± 1.26	2.14 ± 0.95	12.53 ± 4.11
Middle levels of e-health literacy	249	13.56 ± 4.32	5.36 ± 1.64	2.54 ± 1.05	19.84 ± 4.21
High levels of e-health literacy	135	17.21 ± 3.96	5.98 ± 1.52	2.88 ± 1.24	24.17 ± 3.54
*p*		<0.001	<0.001	<0.001	<0.001

The comparison of general data among the three eHL groups (low, middle, and high) revealed significant differences in several variables. Education level (*p* = 0.034), average monthly household income (*p* < 0.001), living alone (*p* < 0.001), dialysis duration (*p* < 0.001), number of complications (*p* < 0.001), and self-management abilities (*p* < 0.001) showed statistically significant variations across the groups. Patients with high eHL tended to have higher education levels (42.0% with college or above) and average monthly household income (4634.85 Yuan), and were more likely to live alone (39.6%). They also had longer dialysis duration (9.51 ± 3.16 years), more complications (6.19 ± 1.59), and lower self-management abilities (15.7%). In contrast, no significant differences were observed in sex (*p* = 0.411), age (*p* = 0.222), smoking (*p* = 0.519), drinking (*p* = 0.171), marital status (*p* = 0.275), employment status (*p* = 0.142), healthcare payment methods (*p* = 0.541), or dialysis frequency (*p* = 0.316). These findings highlight the demographic and clinical factors associated with varying levels of eHL among MHD patients (Table 3).

**Table 3:** Comparison of the general data among the three groups (*n* = 500).

Variables	Low levels of e-health literacy (*n* = 116)	Middle levels of e-health literacy (*n* = 249)	High levels of e-health literacy (*n* = 135)	*p*
Sex, *n* (%), Male	48 (41.6)	114 (45.8)	65 (47.9)	0.411
Age, (Years)	45.12 ± 14.73	47.65 ± 12.49	46.39 ± 11.18	0.222
Smoking, *n* (%)	21 (18.1)	43 (17.3)	18 (13.3)	0.519
Drinking, *n* (%)	36 (31.0)	58 (23.3)	29 (21.5)	0.171
Education level, *n* (%)				**0.034**
Middle school or below	25 (21.4)	88 (35.4)	58 (43.2)	
High school or vocational school	45 (38.5)	116 (46.7)	6 (14.8)	
College or above	46 (40.1)	45 (17.9)	71 (42.0)	
Marital Status, *n* (%)				0.275
Married	96 (82.5)	196 (78.6)	116 (86.3)	
Others	20 (17.5)	53 (21.4)	19 (13.7)	
Average Monthly Household Income (Yuan)	2926.39 (2547.87,3587.24)	3874.17 (2986.37,4336.49)	4634.85 (3784.56,6438.55)	**<0.001**
Employment status, *n* (%), Yes	49 (29.8)	79 (31.7)	46 (31.5)	0.142
Living Alone, *n* (%)	15 (12.5)	60 (24.1)	53 (39.6)	**<0.001**
Healthcare Payment Methods, *n* (%)				0.541
Rural Cooperative Medical Scheme	69 (59.7)	154 (61.8)	79 (58.4)	
Medical health insurance	37 (32.3)	74 (29.8)	45 (33.1)	
Others	10 (8.0)	21 (8.4)	11 (8.5)	
Dialysis Duration (Years)	4.62 ± 2.15	7.21 ± 2.86	9.51 ± 3.16	**<0.001**
Dialysis Frequency every week, *n* (%)				
<3	14 (12.3)	24 (9.8)	14 (10.7)	0.316
≥3	102 (87.7)	225 (90.2)	121 (89.3)	
Complications	2.12 ± 0.58	4.34 ± 0.83	6.19 ± 1.59	**<0.001**
Self-management abilities, *n* (%), Yes	65 (56.3)	96 (38.6)	21 (15.7)	**<0.001**

*P* < 0.05 in bold type.

Multivariate logistic regression analysis was performed to identify factors associated with different levels of eHL (low, middle, and high) among MHD patients. Compared to the low eHL group (reference), patients with a college or above education level were significantly more likely to belong to the high eHL group (OR = 1.512, 95% CI: 1.214–2.263, *p* = 0.023). A higher average monthly household income (>5,000 Yuan) was associated with increased odds of middle eHL (OR = 1.511, 95% CI: 1.213–2.347, *p* = 0.034). Longer dialysis duration (>5 years) was significantly linked to higher eHL (OR = 1.314, 95% CI: 1.086–2.157, *p* = 0.019). Additionally, patients with self-management abilities were more likely to be in the middle eHL group (OR = 1.243, 95% CI: 1.057–2.341, *p* = 0.042). However, living alone, complications, and income levels between 3,000–5,000 Yuan did not show significant associations with eHL levels (*p* > 0.05). These findings suggest that higher education, greater income, longer dialysis duration, and self-management abilities are key factors influencing eHL levels in this patient population (Table 4).

**Table 4:** Multivariate logistic regression of latent profiles for e-health literacy among these patients.

Variables	M vs. L			H vs. L		
	B	*p*	OR (95%Cl)	B	*p*	OR (95%Cl)
Education						
Middle school or below			Reference			Reference
High school or vocational school	0.256	0.341	1.247 (0.546 ~ 1.349)	0.196	0.276	1.247(0.523 ~ 1.616)
College or above	0.451	0.062	1.389(0.556 ~ 1.886)	0.512	**0.023**	1.512(1.214 ~ 2.263)
Average Monthly Household Income (Yuan)						
< 3,000			Reference			Reference
3,000 ~ 5,000	0.312	0.186	1.458 (0.375 ~ 1.824)	0.285	0.105	1.348(0.527 ~ 1.531)
>5,000	0.423	**0.034**	1.511 (1.213 ~ 2.347)	0.537	0.067	1.257(0.582 ~ 1.623)
Living alone						
Yes	−0.241	0.214	0.821 (0.543 ~ 1.241)	−0.325	0.314	0.682 (0.445 ~ 1.157)
No			Reference			Reference
Dialysis duration (Years)						
≤5			Reference			Reference
>5	0.314	0.127	1.243 (0.529 ~ 1.693)	0.412	**0.019**	1.314(1.086 ~ 2.157)
Complications						
≤3	0.283	0.247	1.321 (0.854 ~ 1.952)	0.364	0.086	1.519 (0.892 ~ 1.878)
>3			Reference			Reference
Self-management abilities						
Yes	0.513	**0.042**	1.243 (1.057 ~ 2.341)	0.342	0.104	1.384(0.852 ~ 2.017)
No			Reference			Reference

M, Middle levels of e-health literacy; L, Low levels of e-health literacy; H, High levels of e-health literacy; OR, odd ratio; C, Confidence Interval.*P* < 0.05 in bold type.

The comparison of health-promoting lifestyle scores among MHD patients with different levels of eHL revealed significant differences across all dimensions and total scores (*p* < 0.001). Patients with high eHL (C1, *n* = 135) scored the highest in interpersonal relationships (17.14 ± 2.89), nutrition (26.71 ± 4.09), health responsibility (24.16 ± 5.87), physical activity (15.31 ± 5.14), stress management (18.14 ± 6.23), mental growth (17.94 ± 6.17), and total scores (119.58 ± 13.86). In contrast, patients with middle eHL (C2, *n* = 249) demonstrated moderate scores across all dimensions, with a total score of 103.84 ± 12.72. Patients with low eHL (C3, *n* = 116) scored the lowest in interpersonal relationships (14.87 ± 3.09), nutrition (22.87 ± 4.53), health responsibility (19.78 ± 5.61), physical activity (10.85 ± 3.86), stress management (14.89 ± 5.67), mental growth (14.16 ± 5.93), and total scores (91.82 ± 11.73). These findings indicate a clear gradient in health-promoting lifestyle behaviors, with higher eHL associated with better overall health-promoting practices (Table 5).

**Table 5:** Comparison of total and sub-scale scores of health-promoting lifestyle among different categories of maintenance hemodialysis patients (x ± s).

Number of examples	Categories	Interpersonal relationship	Nutrition	Health responsibility	Physical activity	Stress management	Mental growth	Total scores
135	High levels of e-health	18.45 ± 3.16	27.38 ± 4.12	24.16 ± 5.87	15.31 ± 5.14	18.14 ± 6.23	17.94 ± 6.17	119.58 ± 13.86
249	Middle levels of e-health	14.89 ± 3.07	24.51 ± 3.89	22.41 ± 5.34	12.84 ± 4.27	16.41 ± 5.59	15.13 ± 5.89	103.84 ± 12.72
116	Low levels of e-health	12.94 ± 8.96	21.57 ± 3.91	19.78 ± 5.61	10.85 ± 3.86	14.89 ± 5.67	14.16 ± 5.93	91.82 ± 11.73
*p*-value	*p*	<0.001	<0.001	<0.001	<0.001	<0.001	<0.001	<0.001

## 4 Discussion

This study identified three distinct latent profiles of eHL among MHD patients—low, middle, and high—using LPA. A clear gradient was observed, wherein higher eHL was significantly associated with superior scores across all dimensions of HPL. Multivariate analysis identified higher education, greater household income, longer dialysis duration, and self-management abilities as significant predictors of eHL classification. This categorization is consistent with research conducted among other populations with chronic diseases, as well as community-residing older adults, where eHL levels were likewise divided into clearly defined subgroups (14, 15). This greatly enhances the feasibility and effectiveness of subsequent targeted interventions.

The low eHL group scored poorly across all dimensions, suggesting limited ability to navigate online health resources. In contrast, the high-literacy group demonstrated superior skills in applying and critically evaluating health information. For MHD patients, who require continuous self-management, such disparities in eHL may exacerbate existing health inequities (16). For instance, low-literacy patients may struggle to adhere to fluid restrictions or medication regimens due to inadequate access to or misinterpretation of online guidelines.

An intriguing and seemingly paradoxical finding was that those MHD patients with more complications tended to have higher eHL. This likely does not indicate that eHL causes complications, but rather reflects a stronger motivation for information-seeking among more severely ill patients. Faced with greater health threats and uncertainty, these individuals may become more proactive in utilizing online resources to understand and cope with their disease. This finding aligns with theories of illness perception, where greater perceived threat can motivate more active coping strategies, including information-seeking (17). However, this also presents a nuance: high eHL can be a response to high health needs and may not always correlate with positive outcomes if the information sought is of poor quality or leads to anxiety. This highlights the complex, multifaceted nature of eHL and underscores the necessity of guiding patients toward credible resources and integrating digital literacy with structured education.

Patients with college education or higher were 1.5 times more likely to belong to the high-literacy group, consistent with prior research linking education to digital literacy (18). Higher education likely enhances problem-solving skills and familiarity with technology, enabling patients to navigate complex health information systems. Similarly, higher household income correlated with middle literacy levels, possibly reflecting greater access to digital devices and internet services. Moreover, our study also revealed that groups with higher average monthly household income levels exhibited correspondingly greater eHL proficiency, which aligns with the findings reported by Nelson LA et al. in their research (19).

Longer dialysis duration was associated with higher eHL, suggesting that prolonged exposure to healthcare systems motivates patients to engage with digital resources. Over time, MHD patients may develop “health information-seeking routines” to manage complications or optimize treatment outcomes (20). This represents a learning process akin to “long illness makes a doctor of the patient. Conversely, self-management abilities showed a counterintuitive inverse relationship: patients with lower self-management skills were more likely to fall into the middle-literacy group (21). Clinicians should thus emphasize the integration of digital literacy with structured self-management training to mitigate misinformation risks. Counterintuitive” finding (worse self-management in the meddle literacy group). We propose a plausible explanation: these patients, potentially due to poorer self-management skills and lower confidence in their own care, may become more “dependent on” or “misuse” online information as a substitute for professional guidance. This raises a critical warning: higher eHL does not always equate to correct behaviors and must be integrated with structured self-management training.

The relationship between eHL and self-management behavior is not merely a direct correlation but a complex process involving multiple mediating factors. Table 3 shows that self-management abilities differed significantly among the three groups, with the high eHL group having the highest proportion of patients reporting self-management abilities. This directly demonstrates the strong association between eHL and self-management behavior. The multivariate analysis in Table 4 further indicates that self-management ability is an independent predictor of eHL classification after controlling for other variables. Table 5 shows that the high eHL group scored highest on the Health Responsibility dimension of the HPLP-II, which directly reflects the core of self-management behavior. In summary, eHL is not directly equivalent to self-management behavior but functions through a comprehensive “Knowledge-Empowerment” mechanism (22).

The study demonstrates a strong gradient in HPL scores across eHL classes. These results align with international evidence linking eHL to proactive health behaviors, such as dietary adherence and exercise (23, 24). Effective information seeking is itself a “stress management” and “spiritual growth” strategy. For MHD patients, improved HPL may translate to reduced cardiovascular complications, better fluid control, and enhanced QoL. Notably, the high-literacy group excelled in health responsibility—a dimension encompassing adherence to medical advice and preventive practices. Access to credible online resources empowers patients to monitor laboratory results, understand dialysis adequacy, and recognize early signs of complications like hyperkalemia or infection (25). Conversely, low-literacy patients scored poorly in mental growth and stress management, indicating that limited digital engagement may exacerbate psychological distress. This aligns with studies showing that eHL buffers against anxiety by providing patients with actionable coping strategies (26).

The stratification of eHL into three classes offers a framework for personalized interventions. For the low-literacy group, targeted strategies should focus on foundational digital skills, such as using health apps or verifying online information. Healthcare providers could collaborate with community centers to offer workshops on basic internet navigation and critical appraisal of health content. For middle-literacy patients, interventions should emphasize advanced skills, such as interpreting lab results or communicating with clinicians via telehealth platforms. The high-literacy group may benefit from peer-led initiatives, where tech-savvy patients mentor others in leveraging online tools for self-care. Furthermore, integrating eHL assessments into routine clinical practice could identify at-risk patients. Clinical intervention strategies for each identified subgroup, transforming them from vague suggestions into practically executable plans. Nurses and nephrologists could use these assessments to tailor educational materials—for example, simplifying dietary guidelines for low-literacy patients or curating reputable websites for high-literacy individuals.

From the perspectives of Social Cognitive Theory and Empowerment Theory, the positive relationship between high eHL and health-promoting behaviors in MHD patients may be understood as a process wherein eHL contributes to self-efficacy and empowerment. Our findings are highly consistent with these theoretical principles. Successfully obtaining and applying online information to solve health problems significantly enhances patients’ self-efficacy. According to Bandura’s Social Cognitive Theory, high self-efficacy is a key driver of behavior change (27). High eHL is essentially an empowerment process (28). Our results gain further significance when contextualized within healthcare delivery systems, where eHL stratification emerges as a valuable tool for resource planning and care intensity interpretation. The demonstrated association between low eHL and increased healthcare utilization suggests that measuring eHL could help identify patients requiring more intensive support services, thereby enabling more efficient allocation of limited resources. This approach aligns with emerging evidence linking health literacy to nursing care complexity and overall service delivery demands (29, 30). Particularly in resource-constrained settings, understanding the literacy landscape of patient populations can inform the development of tailored interventions that maximize impact while minimizing unnecessary expenditure.

While this study advances understanding of eHL in MHD patients, several limitations warrant attention. First, the cross-sectional design precludes causal inferences. Longitudinal studies are needed to explore how eHL evolves over time and influences long-term outcomes like hospitalization rates or survival. Most importantly, the cross-sectional nature of our data prohibits any causal inferences. While we identified a strong association between eHL profiles and HPL, and controlled for key confounders, the relationship may be influenced by unmeasured confounding variables. Therefore, our findings should be interpreted as revealing a robust predictive association rather than a causal effect. Second, the sample was restricted to three tertiary hospitals in Baoding City, China, limiting generalizability to rural or low-resource settings. Future research should include diverse geographic and socioeconomic populations. In particular, multi-center studies involving participants from different regions and social strata are strongly recommended to validate the stability and broader applicability of the identified latent profiles. Third, self-reported measures are susceptible to social desirability bias. Objective metrics, such as step counts or biochemical markers, could complement self-assessment in future studies. Lastly, the study did not explore the role of caregivers or healthcare providers in mediating eHL. Family support and clinician-patient communication may significantly shape digital health behaviors.

## 5 Conclusion

This study highlights the value of eHL in relation to HPL among MHD patients. By identifying distinct latent profiles, we highlight the need for stratified interventions that address the unique challenges of low-, middle-, and high-literacy groups. Enhancing digital competencies in this vulnerable population may not only improve self-management but also reduce the burden on healthcare systems. As dialysis care increasingly incorporates telehealth and remote monitoring, fostering eHL must become a cornerstone of holistic nephrology practice. Future efforts should prioritize equity, ensuring that all patients—regardless of socioeconomic status—can harness the power of digital health to achieve better outcomes.

## Data Availability

The raw data supporting the conclusions of this article will be made available by the authors, without undue reservation.
